# Blood flow in the optic nerve head in patients with primary aldosteronism

**DOI:** 10.1371/journal.pone.0285039

**Published:** 2023-04-26

**Authors:** Kazuyuki Hirooka, Kenji Oki, Keiko Ogawa-Ochiai, Yuta Nakaniida, Hiromitsu Onoe, Yoshiaki Kiuchi

**Affiliations:** 1 Department of Ophthalmology and Visual Science, Hiroshima University, Hiroshima, Japan; 2 Department of Molecular and Internal Medicine, Hiroshima University, Hiroshima, Japan; 3 Kampo Clinical Center, Hiroshima University Hospital, Hiroshima, Japan; Doheny Eye Institute/UCLA, UNITED STATES

## Abstract

**Purpose:**

Optic nerve head (ONH) blood flow decrease without changes in intraocular pressure in a possible rat model of retinal ganglion cell loss by systemic administration of aldosterone. To compare the blood flow in the ONH, using laser speckle flowgraphy (LSFG), in healthy eyes and in eyes with primary aldosteronism (PA).

**Methods:**

The ONH tissue area mean blur rate (MT) was evaluated in this single center, retrospective, cross-sectional study using LSFG. In order to compare the MT between PA patients and normal subjects, mixed-effects models were used, with adjustments made for the mean arterial pressure, disc area, and β-peripapillary atrophy (β-PPA) area. Mixed-effects models were also used to analyze the risk factors affecting the MT.

**Results:**

This study evaluated a total of 29 eyes of 17 PA patients and 61 eyes of 61 normal subjects. There was a significantly lower MT in PA patients (10.8 ± 0.4) as compared to the normal subjects (12.3 ± 0.3) (*P* = 0.004). The MT was significantly lower in PA patients (10.8 ± 0.6) even after adjusting for the potential confounding factors when compared to normal subjects (12.3 ± 0.3) (*P* = 0.046). Multivariate mixed-effects model analysis demonstrated that the MT was significantly associated with the PA and β-PPA.

**Conclusions:**

There was a significantly lower ONH blood flow in PA patients as compared to normal subjects.

## Introduction

Aldosterone exerts its effects on sodium and fluid homeostasis via binding to the mineralocorticoid receptor (MR). Aldosterone has recently been investigated as a contributor to the various deleterious effects of ocular diseases, such as diabetic retinopathy, retinal vein occlusion, and central serous chorioretinopathy [1]. Due to tumors or hyperplasia, there is an excess production of aldosterone from the adrenal glands, with the subsequent elevated plasma aldosterone concentrations in patients with primary aldosteronism (PA) found to be the most common form of secondary hypertension. Therefore, PA patients may have a higher risk for vision loss due to various ocular diseases.

Determination of ocular blood flows can be achieved through the use of a non-contact technique, laser speckle flowgraphy (LSFG) [2]. The quantitative index of the blood flow velocity in target tissues is defined as the mean blur rate (MBR), which can be determined by the current LSFG instruments. There are two MBRs that can be measured for the ONH, with the ONH tissue-area MBR (MT) indicating the ONH microcirculation, while the ONH vessel-area MBR (MV) showing the blood velocity through the large visible vessels present in the ONH area [3–5]. In order to determine interindividual comparisons, previous studies have shown that the MT can be used for these analyses [6,7]. Moreover, other studies have suggested that a quantitative index of the ONH tissue blood flow in humans can potentially be determined based on the MT. Results of these previous studies have shown that glaucomatous eyes exhibit a reduced MT, with the extent of this decrease being dependent on the severity of the glaucoma [8].

It has also been reported that there is a decrease in the number of retinal ganglion cells (RGCs), without any changes in other retinal layers or the IOP after a systemic administration of aldosterone [9]. Moreover, plasma aldosterone concentration increases have been observed after a systemic administration of aldosterone, with the relationship between the number of RGCs and the plasma aldosterone concentration exhibiting a negative correlation [10]. LSFG measurements have additionally documented that there were significant decreases in the MT after systemic administration of aldosterone [11].

The purpose of the current study was to evaluate healthy eyes and eyes with PA using LSFG and then compare the ONH blood flows.

## Materials and methods

### Subjects

Between October 2021 and April 2022, this retrospective cross-sectional study was conducted at Hiroshima University Hospital. The Institutional Review Board of the Hiroshima University approved this study protocol (E2006-0634). In accordance with the principles outlined in the Declaration of Helsinki, all subjects provided written informed consent.

The Japan Endocrine Society 2009 guidelines for the diagnosis and treatment of PA were used to evaluate and define PA in the enrolled patients in this study [12]. Briefly, in order to confirm a diagnosis of PA in patients, after screening for an aldosterone-to-renin ratio [ARR; plasma aldosterone concentration (pg/ml)/plasma renin activity (ng/ml per h)] of more than 200, subjects then underwent a captopril-challenge test, upright furosemide-loading test, and saline-loading test. Subsequently, all PA patients underwent measurement of PAC and plasma cortisol concentrations in adrenal venous blood using an adrenal vein sampling technique under adrenocorticotropic hormone stimulation in order to identify the lateralization of the aldosterone secretion.

A comprehensive ophthalmological screening examination was also performed in all patients, with the tests including best corrected visual acuity (BCVA), refraction using an auto-refractometer (ARK-730, Topcon), IOP using a Goldmann applanation tonometer, central corneal thickness using a specular microscope (SP-2000, Topcon), axial length using the IOL Master (Carl Zeiss Meditec, Dublin, CA), slit-lamp biomicroscopic examination, gonioscopy, ophthalmoscopy, fundus photography, and optical coherence tomography (OCT; RTVue-XR Avanti, Optovue Inc. Fremont, CA). Eyes that exhibited structural glaucomatous changes, vertical cup-disc asymmetry between fellow eyes of ≥ 0.2, a cup-to-disc ratio of ≥ 0.7, and neuroretinal rim narrowing, notches, localized pallor, or retinal nerve fiber layer defects were defined as being glaucomatous eyes. Patients that had a best-corrected visual acuity of <20/40, spherical refractive errors exceeding ± 6.0 diopters (D), refractive cylindrical errors of > 2.0 D, axial length of ≥ 27 mm, IOP of > 21 mmHg, any abnormal findings in the fundus, any findings suggestive of the presence of glaucoma or an anomaly in the disc and peripapillary retina, history of retinal diseases and intraocular surgery, history of smoking, and history of diabetes mellitus were excluded from the study. For all of the normal subjects, we also excluded any patients with systemic hypertension. Systemic hypertension was defined as a current use of antihypertensive medicine and/or systolic blood pressure (SBP) ≥ 140 mmHg and/or diastolic blood pressure (DBP) ≥ 90 mmHg [13]. If both eyes met the criteria in normal subjects, the right eye was selected.

### Laser speckle flowgraphy

A LSFG (LSFG-NAVI; Software Ltd., Fukuoka, Japan) was used to measure the ONH blood flow. The principles of LSFG have been previously described [2]. The instrument is equipped with a diode laser (wavelength; 830 nm) and an ordinary charge-coupled device camera (resolution; 750 × 360 pixels) that can be used for obtaining images of the fundus. Measurements are based on the speckle contrast production pattern that is created due to the interference of a laser by moving blood cells in the blood vessels. The ONH area is automatically divided into the large visible vessels and capillary area by the accompanying LSFG software, in addition to providing the MT, MV, and all-area MBR as the sum of the MT and MV. As our previous study demonstrated that the MT measurement reflects the capillary blood flow and can be used for intergroup comparisons, our current study specifically focused on the MT variable [7]. The measurements in each eye were repeated 3 times, with the statistical analysis using the mean values of the MT for the comparisons. Immediately after the LSFG measurements, the SBP and DBP were analyzed. In order to calculate the mean arterial pressure (MAP), the following equation was used: MAP = DBP + 1/3 (SBP–DBP), with the results obtained as described below.

### β-PPA area measurements

For the β-peripapillary atrophy (β-PPA) area measurements, the OCT-determined disc area was first inspected, and corrected as needed, by viewing the Bruch’s membrane opening (BMO) positions in the B-scan images. The clinical disc margin on the scanning laser ophthalmoscopy image was then determined with reference to the fundus photo, which was presented in another display. Subsequently, the circle was again modified in order to match the outer border of the β-PPA, with altered values of the OCT-determined disc area then automatically shown. Subtracting the BMO area from the area inside the outer border of the β-PPA yielded the β-PPA area.

### Statistical analysis

The mixed-effects model analysis for numerical data, which accounted for the correlations between the fellow eyes in the same patient, was performed for comparisons between the PA group and normal subject group. After adjusting for the MAP, disc area and β-PPA area, eyes of the PA patients were compared to normal subjects for the MT by using a multivariate general linear model [14]. Factors affecting the MT were determined through the use of a multivariate linear mixed-effect modeled regression analysis. Age, gender, MAP, IOP, axial length, disc area, cup area and β-PPA area were defined as the independent variables. JMP software version 16 (SAS Inc., Cary, NC) was used for all of the statistical analyses. A *P* value less than 0.05 was considered statistically significant. All of the data are presented as the mean ± standard error of the mean.

## Results

This study evaluated a total of 29 eyes of 17 PA patients and 61 eyes of 61 normal subjects. The demographic and ocular characteristics of the study participants are summarized in Table 1. The mean age in the normal subjects and in the PA patients was 51.1 ± 1.5 years and 52.3 ± 2.1 years, respectively (*P* = 0.40). Neither the axial length (24.6 ± 0.2 mm and 24.5 ± 0.3 mm, respectively, *P* = 0.84) nor the β-PPA (0.35 ± 0.6 mm^2^ and 0.30 ± 0.09 mm^2^, respectively, *P* = 0.64) differed between the normal subjects and the PA patients. There was a significantly reduced IOP (12.0 ± 0.4 mmHg), disc area (1.96 ± 0.06 mm^2^), and cup area (0.70 ± 0.05 mm^2^) in the normal subjects as compared to the PA patients (14.2 ± 0.5 mmHg, 2.36 ± 0.08 mm^2^, 0.95 ± 0.07 mm^2^, respectively), all *P* < 0.01.

**Table 1:** Clinical characteristics of normal subjects and primary aldosteronism patients.

	Normal subject	Primary aldosteronism	*P* value
No. eyes/individuals	61/61	29/17	
Age (years)	51.1 ± 1.5	53.3 ± 2.1	0.40
Gender (M/F)	13/49	7/10	0.10
SBP (mmHg)	107.0 ± 2.2	134.1 ± 4.0	<0.001
DBT (mmHg)	62.9 ± 1.6	84.8 ± 2.8	<0.001
MAP (mmHg)	77.6 ± 1.8	101.0 ± 2.5	<0.001
IOP (mmHg)	12.0 ± 0.4	14.2 ± 0.5	0.001
Axial length (mm)	24.6 ± 0.2	24.5 ± 0.3	0.84
Disc area (mm^2^)	1.96 ± 0.06	2.36 ± 0.08	<0.001
Cup area (mm^2^)	0.70 ± 0.05	0.95 ± 0.07	0.003
β-PPA area (mm^2^)	0.35 ± 0.06	0.30 ± 0.09	0.64

M; male, F; female, SBP; systolic blood pressure, DBT; diastolic blood pressure.

MAP; mean arterial pressure, IOP; intraocular pressure, PPA; peripapillary atrophy.

The MT was 12.3 ± 0.3 and 10.8 ± 0.4 in normal subjects and PA patients, respectively (*P* = 0.004) prior to adjusting for the MAP, disc area and β-PPA area. After adjusting for the MAP, disc area and β-PPA area, there was a significant difference observed for the MT between the PA patients and normal subjects (Table 2) (*P* = 0.046).

**Table 2:** Adjusted MT in eyes with normal subject and primary aldosteronism.

	Normal subject	Primary aldosteronism	*P* value
MT (AU)	12.3 ± 0.3	10.8 ± 0.6	0.046

MT; tissue-area mean blur rate, AU; arbitary unit.

In order to identify the factors that affected the MT, multiple regression analysis was performed (Table 3). Results showed that there was a significant association between PA (β = 0.670, *P* = 0.01) and β-PPA (β = -1.567, *P* = 0.002) and the MT.

**Table 3:** Stepwise multiple regression analysis for factors associated MT.

		Univariate			Multivariate	
Factors	β	SE (β)	*P* value	β	SE (β)	*P* value
Normal subject	0.790	0.268	0.004	0.670	0.263	0.01
Age (years)	-0.048	0.023	0.04	-0.033	0.021	0.12
Gender (F)	0.801	0.284	0.006			
MAP (mmHg)	-0.035	0.015	0.02			
IOP (mmHg)	0.008	0.088	0.92			
Axial length (mm)	-0.165	0.194	0.40			
disc area (mm^2^)	-0.814	0.558	0.14			
cup area (mm^2^)	-1.981	0.666	0.004	-0.976	0.658	0.14
β-PPA area (mm^2^)	-1.521	0.541	0.006	-1.567	0.491	0.002

MT; tissue-area mean blur rate, F; female, MAP; mean arterial pressure.

IOP; intraocular pressure, PPA; peripapillary atrophy.

**Fig 1:**
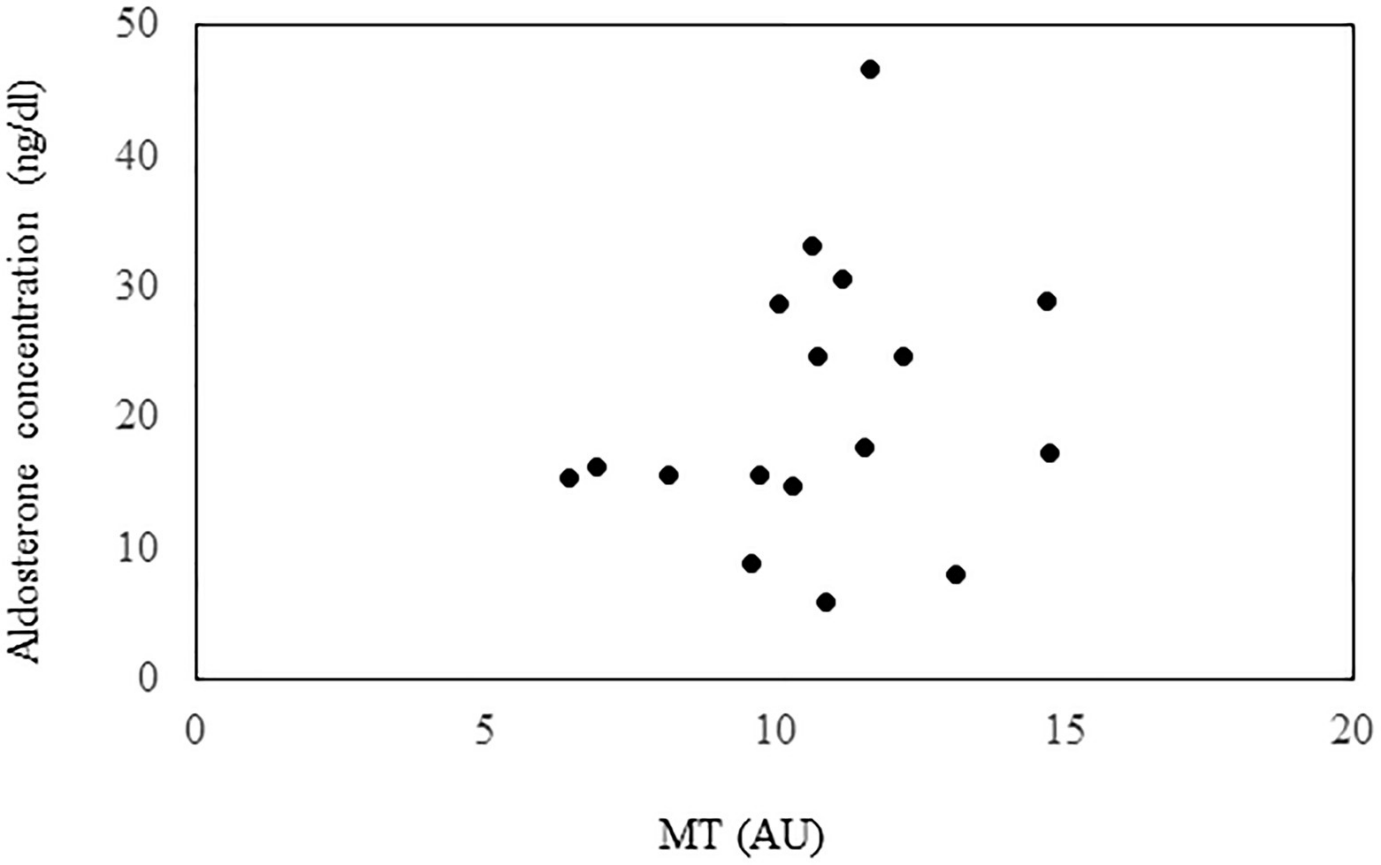
Scatterplots showing the relationship between MT and plasma aldosterone concentration.

Subsequently, the PA patients were investigated for the relationship between the plasma aldosterone concentration and the MT. When the criteria were met for both eyes, the right eye was selected. The plasma aldosterone concentration and MT did not exhibit any association (Fig 1; r = 0.224, *P* = 0.39).

## Discussion

Results of the current study demonstrated that there was a significantly decreased MT in PA patients as compared to normal subjects. Furthermore, both PA and β-PPA were shown to be significantly associated with MT by the multiple regression analysis.

In the retina, MRs are expressed in retinal and choroidal vessels and in the retinal pigment epithelium (RPE) cells [15,16]. It is speculated that overactivation of the retina/choroidal MR pathway could contribute to the mechanisms of central serous chorioretinopathy (CSC) [17]. CSC is a part of the pachychoroid spectrum disorders, and is characterized by serous retinal detachment, RPE alterations and dilation of the choroidal vessels. Retinal blood flow velocity and blood flow rate were reported to be lower in eyes with CSC as compared to healthy eyes [18]. Therefore, it has been speculated that MR pathway overactivation may reduce retinal blood flow.

It has been previously reported that changes via acute, non-genomic and chronic genomic effects that subsequently modulate the vascular resistance and blood flow, can be caused by aldosterone [19]. Characteristics of aldosterone-induced vasculopathy include a reduction of endothelial nitric oxide synthesis (eNOS), which catalyzes the synthesis of NO in order to regulate endothelial function, and bioavailability along with an increased generation of superoxide radicals that degrade eNOS [19]. Moreover, aldosterone-induced gene expression of endothelin 1, which is a potent vasoconstrictor secreted by vascular endothelial cells, has been shown to be associated with the activation of MRs [20]. Blood flow changes have additionally been reported to occur due to the administration of aldosterone in experimental animal models and in humans. Fujita et al [21]. examined the left anterior descending coronary artery in open chest dogs and reported finding that an acute aldosterone infusion led to a decreased blood flow. Romagni et al [22]. reported there was a rapid decreased forearm blood flow effect associated with an aldosterone infusion into the antecubital vein of the arm in humans. Moreover, after a systemic administration of aldosterone in rats, there was a decrease in the ONH blood flow without changes in the IOP or SBP [11]. Although PA patients exhibited a significantly higher MAP as compared to the normal subjects, it is our hypothesis that the MT reduction likely reflects the local effects of aldosterone, as our analysis was conducted after adjusting the MAP.

Results for our current study demonstrated that there was a significant negative correlation between the MT and the β-PPA. Recently a significant positive correlation was reported between β-PPA and MT by Anraku et al [14]. However, it should be noted that they only included normal healthy subjects. In our current study, PA patients who took anti-hypertensive medicines were included and thus, this could have affected the blood flow. Therefore, the inclusion of these subjects could have potentially affected the results. Studies that have evaluated normal Japanese subjects have shown that there were no significant impacts on the MT for subjects with a mean age of 39.3 [4], 45 [5], or 48 years [14] of age. In contrast, studies that have examined normal Japanese subjects with a mean age of 63.5 years [23] and normal European white subjects with a mean age of 48.9 years [3] reported that a significant negative correlation was founded between age and MT. In normal Japanese subjects aged less than 50 years, age has little effect on the MT, with the negative effect on the MT only evident in those aged more than 60 years. The mean ages for the normal subjects and PA patients in the current study were 51.1 and 53.3 years, respectively. Because of the similarity in the ages of our subjects, our current study did not adjust for the age. However, after further adjusting for age, we found that there were similar results (normal subject; 12.3 ± 0.3, PA; 10.8 ± 0.5, *P* = 0.043).

Our current study also showed that there was no association between plasma aldosterone concentration and MT. Although a previous experimental model reported that aldosterone led to a decrease in the blood flow, the degree of the increase in the plasma aldosterone concentration was not related to the MT changes [11]. Moreover, our results are supported by a previous animal study. Therefore, although the plasma aldosterone concentration does not appear to be involved, if it does exceed its almost constant level, it may have a similar role.

Previous reports have also shown that there was a significant decrease in the number of RGCs [9] and a significant decrease in the MT after a systemic administration of aldosterone in rats [11]. The lamina cribrosa, which is thought to be the primary site of the lesion in glaucoma, is supplied by the deep ONH blood flow, which is reflected by the MT [24]. Therefore, glaucoma prevalence in PA patients is important and should be investigated. Our research group at the present time is currently conducting additional studies that are designed to investigate PA patients and the prevalence of glaucoma among these patients.

The mean IOP in the normal subjects and in the PA patients was 12.0 ± 0.4 mmHg and 14.2 ± 0.5 mmHg, respectively. The mean IOP in all subjects, including high tension glaucoma, in the Tajimi Study was 14.6 ± 2.7 mmHg in the right eye and 14.5 ± 2.7 mmHg in the left eye [25], which is higher than that found for the normal subjects in our current study. Therefore, the low average IOP that was found in the study population appears to be a contributing factor in our current study.

There were some limitations for our current study. First, the intake of oral anti-hypertensive medications that could affect the blood flow, such as a calcium antagonist α-blocker, or a β-blocker, could have been taken by our PA patients prior to being enrolled in our study. It also should be noted that an increased blood velocity in the ONH has been reported in healthy humans after the administration of lomerizine, which is a calcium antagonist [26]. The use of topical carteolol, which is a β-blocker, was reported by Tamaki et al. [27] to increase the ONH blood velocity in humans. Therefore, these previous findings suggest that there might be a greater decrease in the MT if anti-hypertensive medications were not taken by the PA patients. The second limitation of this study was that there were only a relatively small number of cases evaluated, especially for the PA patients. Therefore, a larger number of PA patients will need to be analyzed in order to more definitively investigate this issue.

## Conclusions

In conclusion, after adjusting for the MAP, disc area and β-PPA area, there was a significantly lower ONH blood flow found in PA patients as compared to that found for normal subjects.

## Supporting information

S1 File (XLSX)
